# Thresholds for the cost–effectiveness of interventions: alternative approaches

**DOI:** 10.2471/BLT.14.138206

**Published:** 2014-12-15

**Authors:** Elliot Marseille, Bruce Larson, Dhruv S Kazi, James G Kahn, Sydney Rosen

**Affiliations:** a Health Strategies International, 555 Fifty-ninth Street, Oakland, California, 94609, United States of America (USA).; b Center for Global Health and Development, Boston University, Boston, USA.; c Division of Cardiology, San Francisco General Hospital, San Francisco, USA.; d Institute for Health Policy Studies, University of California – San Francisco, San Francisco, USA.

## Abstract

Many countries use the cost–effectiveness thresholds recommended by the World Health Organization’s Choosing Interventions that are Cost–Effective project (WHO-CHOICE) when evaluating health interventions. This project sets the threshold for cost–effectiveness as the cost of the intervention per disability-adjusted life-year (DALY) averted less than three times the country’s annual gross domestic product (GDP) per capita. Highly cost–effective interventions are defined as meeting a threshold per DALY averted of once the annual GDP per capita. We argue that reliance on these thresholds reduces the value of cost–effectiveness analyses and makes such analyses too blunt to be useful for most decision-making in the field of public health. Use of these thresholds has little theoretical justification, skirts the difficult but necessary ranking of the relative values of locally-applicable interventions and omits any consideration of what is truly affordable. The WHO-CHOICE thresholds set such a low bar for cost–effectiveness that very few interventions with evidence of efficacy can be ruled out. The thresholds have little value in assessing the trade-offs that decision-makers must confront. We present alternative approaches for applying cost–effectiveness criteria to choices in the allocation of health-care resources.

## Résumé

De nombreux pays utilisent les seuils de rentabilité recommandés par le projet WHO-CHOICE (Choosing Interventions that are Cost–Effective; en français: « choisir des interventions efficaces au meilleur coût ») de l'Organisation mondiale de la Santé lors de l'évaluation des interventions sanitaires. Ce projet définit le seuil de rentabilité comme étant égal au coût de l'intervention par espérance de vie corrigée de l'incapacité (EVCI) évitée moins trois fois le produit intérieur brut (PIB) annuel du pays par habitant. Les interventions très rentables sont définies comme celles satisfaisant un seuil par EVCI évitée égal à une fois le PIB annuel par habitant. Nous soutenons que le recours à ces seuils réduit la valeur des analyses de rentabilité et qu'il rend ces analyses trop grossières pour qu'elles soient utiles pour la prise de décision dans le domaine de la santé publique. L'utilisation de ces seuils est peu justifiée théoriquement, contourne le classement difficile mais nécessaire des valeurs relatives des interventions applicables localement et néglige l'examen de ce qui vraiment abordable. Les seuils de WHO-CHOICE fixent une limite de rentabilité si basse que très peu d'interventions présentant des preuves d'efficacité peuvent être exclues. Les seuils ont peu de valeur pour évaluer les compromis auxquels les décideurs doivent faire face. Nous présentons des approches alternatives pour l'application des critères de rentabilité aux choix liés à l'allocation des ressources de soins de santé.

## Resumen

Numerosos países utilizan los umbrales de rentabilidad recomendados por el proyecto Elección de intervenciones rentables de la Organización Mundial de la Salud – (WHO-CHOICE) al evaluar las intervenciones de salud. Este proyecto establece el umbral de rentabilidad como el coste de la intervención por año de vida ajustado por discapacidad (AVAD) evitado, que es tres veces inferior al producto interno bruto anual del país (PIB) per cápita. Las intervenciones de rentabilidad elevada se definen como el cumplimiento de un umbral por AVAD evitado equivalente a una vez el PIB per cápita anual. Se arguye que la dependencia de estos umbrales reduce el valor de los análisis de rentabilidad y hace que dichos análisis sean demasiado contundentes para que resulten útiles en la mayoría de las decisiones en el campo de la salud pública. El uso de estos umbrales tiene una justificación teórica insuficiente, elude la clasificación difícil pero necesaria de los valores relativos de las intervenciones aplicables a nivel local y omite cualquier consideración de lo que es realmente asequible. Los umbrales de WHO-CHOICE establecen un límite de rentabilidad tan bajo que son muy pocas las intervenciones de eficacia probada que pueden descartarse. Los umbrales tienen poco valor a la hora de evaluar las ventajas y desventajas a las que los responsables de la toma de decisiones deben enfrentarse. Presentamos enfoques alternativos para la aplicación de los criterios de rentabilidad en las decisiones acerca de la asignación de los recursos de salud.

## ملخص

تستخدم العديد من البلدان عتبات المردودية التي أوصى بها مشروع "اختيار التدخلات عالية المردود التابع لمنظمة الصحة العالمية" (WHO-CHOICE) عند تقدير التدخلات في مجال الصحة. ويحدد هذا المشروع عتبة المردودية على أنها تكلفة التدخل لكل سنة تم تفاديها من سنوات العمر المصححة باحتساب مدد العجز الأقل من ثلاث أضعاف الناتج الإجمالي المحلي السنوي للبلد لكل فرد. ويتم تعريف التدخلات عالية المردود على أنها تلبية العتبة لكل سنة تم تفاديها من سنوات العمر المصححة باحتساب مدد العجز لمرة واحدة من الناتج الإجمالي المحلي السنوي لكل فرد. ونرى أن الاعتماد على هذه العتبات يقلل من قيمة تحليلات المردودية ويجعل مثل هذه التحليلات عديمة الفائدة في معظم حالات اتخاذ القرار في مجال الصحة العمومية. ويستند استخدام هذه العتبات إلى مبرر نظري ضعيف ويتجنب الترتيب الصعب والضروري للقيم النسبية للتدخلات السارية على الصعيد المحلي ويغفل النظر عن النهج معقولة التكلفة بالفعل. وتحدد عتبات WHO-CHOICE عتبة دنيا للمردودية يمكن على أساسها استبعاد بضعة تدخلات ذات بيِّنات على الكفاءة. وتكون للعتبات قيمة قليلة في تقييم عمليات الموازنة التي يتعين على متخذي القرار مواجهتها. ونقدم نهجاً بديلة لتطبيق معايير المردودية على الاختيارات في تخصيص موارد الرعاية الصحية.

## 摘要

许多国家在评估卫生干预措施时使用世界卫生组织WHO-CHOICE（选择具有成本效益的干预措施项目）推荐的成本效益阈值。该项目将成本效益阈值设定为避免单位残疾调整生命年（DALY）的干预措施的成本低于国家年度人均国内生产总值（GDP）三倍。将极具成本效益的干预措施定义为达到以单倍年度人均国内生产总值避免的单位DALY的成本的阈值。我们主张，对这些阈值的依赖减少了成本效益分析的价值，使这种分析太过生硬，以致于对大多数公共卫生领域的决策来说用处不大。使用这些阈值几乎没有理论依据，绕开了做起来很难但又不得不去做的对当地适用干预措施相对价值排名，忽略了对任何有关什么才真正实惠的考虑。WHO-CHOICE阈值为成本效益设定的门槛这样低，以至于为数不多具有效力证据的干预措施也会被排除在外。阈值在评估决策者必须面对的权衡上价值微乎其微。我们提出了医疗资源分配方面的选择上成本效益标准应用的替代方法。

## Резюме

Во многих странах используются пороговые значения эффективности затрат, рекомендованные рабочей программой ВОЗ «Выбор мероприятий, эффективных с точки зрения затрат» (WHO-CHOICE), при оценке проводимых мероприятий в области здравоохранения. Этот проект устанавливает пороговое значение эффективности затрат как стоимость мероприятия на количество предотвращенных лет жизни, утраченных в результате инвалидности (ДАЛИ), не превышающая три годовых валовых внутренних продукта (ВВП) страны на душу населения. При этом высокоэффективными мероприятиями считаются те, которые соответствуют пороговому значению на предотвращенное ДАЛИ в размере, не превышающем одного годового ВВП на душу населения. Мы утверждаем, что использование этих пороговых значений снижает стоимость анализа эффективности затрат и делает подобный анализ поверхностным для большинства случаев принятия решений в области общественного здравоохранения. Для использования этих пороговых значений не имеется достаточных теоретических обоснований, они упускают из виду трудоемкое, но необходимое ранжирование относительной стоимости применяемых локально мероприятий, а также не рассматривают доступность подобных мероприятий. Программой WHO-CHOICE устанавливается такая низкая планка для эффективности затрат, что лишь немногие мероприятия с признаками эффективности могут быть исключены. Эти пороговые значения не имеют большой ценности в процессе принятия компромиссных решений, с которыми приходится иметь дело отвественным лицам. Мы предлагаем альтернативные подходы для применения критериев эффективности затрат при выборе предпочтительных вариантов в процессе распределения ресурсов здравоохранения.

## Introduction

In public health, cost–effectiveness analyses compare the costs and effectiveness of two or more health interventions – with effectiveness measured in the same units. When comparing interventions, the incremental cost–effectiveness ratio (ICER) – i.e. the difference in costs divided by the difference in health effects – is often used to express the result.

Estimates of costs, health effects and ICERs provide clear guidance to policy-makers in three situations: (i) when the health-effect target is specified by policy-makers and the aim of the cost–effectiveness analysis is to minimize the expenditure needed to achieve that target; (ii) when a budget constraint is specified by policy-makers and the aim is to maximize the health benefits while keeping expenditure within budget; and (iii) when policy-makers have specified an explicit standard or threshold for what should be considered cost–effective. In all three cases, the analysts completing the cost–effectiveness analysis cannot objectively make a recommendation to policy-makers without prior decisions by policy-makers on health-effect or cost targets or thresholds. Without reference to such decisions, the cost–effectiveness analysis cannot fully orient policy-makers to the range of options that might be good investments. 

For example, compared with no vaccination, routine quadrivalent human papillomavirus vaccination combined with catch-up vaccination – to protect against cervical diseases in Brazil – was found to have an ICER of 450 United States dollars (US$) per quality-adjusted life-year (QALY) gained.^1^ In the United Republic of Tanzania, compared with no treatment, post-exposure prophylaxis for rabies was found to have an estimated ICER of US$ 27 per QALY gained.^2^ However, how does one decide whether US$ 450 per QALY gained in Brazil or US$ 27 per QALY gained in the United Republic of Tanzania represents good use of money for the national health-care system?

Three general approaches have been used to solve this problem: (i) thresholds based on per capita national incomes; (ii) benchmark interventions and (iii) league tables. In recent years, the most common approach has involved the use of thresholds based on per capita gross domestic product (GDP). Under this approach – which has been promoted by the World Health Organization’s Choosing Interventions that are Cost–Effective (WHO-CHOICE) project^3^ – an intervention that, per disability-adjusted life-year (DALY) avoided, costs less than three times the national annual GDP per capita is considered cost–effective, whereas one that costs less than once the national annual GDP per capita is considered highly cost–effective.

In this article, we argue that the current thresholds based on per capita GDP have major shortcomings as guides for policy-makers, since each of the available approaches has substantial weaknesses. We then discuss that a new consensus should be reached on a process for evaluating the cost–effectiveness of health interventions that places ICERs in the context of other, local policy and programme options, including funding sources. We focus on cost–effectiveness and ignore other criteria for policy decisions, such as equity, ethics and political feasibility. We proceed from the premise that evidence-based economic evaluations are vital additions to public policy decisions – which would otherwise largely reflect political, ideological and/or bureaucratic concerns. We focus on the relative merits of different ways of distinguishing what constitutes an acceptable level of cost–effectiveness and on the limitations of the widely used national-income-based approach.

## Thresholds

The most pervasive threshold was initially promoted by the Commission on Macroeconomics and Health and adopted in *The world health report 2002* and by WHO-CHOICE. This threshold links per capita GDP with returns on investments in health to define the characteristics of a cost–effective and a very cost–effective intervention.^4^^–^^6^ Many published cost–effectiveness analyses of health interventions in low resource countries now explicitly refer to these WHO criteria as the standards by which each intervention is considered cost–effective or not. However, use of these criteria has at least four major limitations.

The first limitation is that important comparisons are obscured. Cost–effectiveness analysis is useful only in the context of the choices available in a particular setting and context – e.g. the budget and technical capacity of a national malaria control programme or Ministry of Health. Even if an intervention is categorized as cost–effective based on its cost per DALY averted, that intervention may still not represent the best use of a country’s health budget (Box 1). It is not enough to know that, per DALY avoided, an intervention costs less than three times the local annual per capita gross domestic product. We also need to know if it costs less – per DALY avoided – than other needed and feasible interventions. The current shift in some of the United States of America’s global health funding – i.e. away from support for the treatment of human immunodeficiency virus (HIV) infections and towards malaria, maternal and child health and other programmes – tacitly recognizes that, even among activities with ICERs below a national-income threshold, trade-offs are real and consequential.

Box 1 **Widely differing cost–effectiveness ratios of programmes considered very cost–effective according to WHO-CHOICE criteria**

In Zambia, three public health strategies have dramatically differing cost–effectiveness ratios compared with doing nothing:

- Expansion of access to insecticide-treated bednets for malaria prevention: this intervention has an estimated cost of 29 international dollars (I$) per disability-adjusted life-year (DALY) averted, so I$ 1 million spent on bednets could avert 34 483 DALYs.^6^
- Screening and treatment of syphilis in pregnancy: depending on the setting, the cost–effectiveness of this intervention ranges from saving money to a cost of I$ 127 per DALY averted.^7^ I$ 1 million spent on this intervention could avert 7859 DALYs.
- Antiretroviral therapy (ART) for patients infected with human immunodeficiency virus: a recent study shows that – compared with cotrimoxazole prophylaxis – this would cost I$ 963 per DALY averted.^8^ I$ 1 million spent on ART could thus avert 1038 DALYs.

All three of these interventions easily meet the WHO-CHOICE threshold for being highly cost–effective; the annual per capita GDP (about I$ 1684 in Zambia) per DALY averted. However, compared with investing I$ 1 million in ART, investing the same amount in syphilis screening and treatment in pregnancy or in bednets would avert 7.6- and 33-fold more DALYs, respectively. Thus simply stating that an intervention is cost–effective by WHO’s standards masks the real trade-offs among competing strategies.

GDP: gross domestic product.

The second limitation is that thresholds are too easily attained. Beyond the virtue of availability, we are puzzled why per capita gross domestic products were chosen as the main units for cost–effectiveness thresholds. Too many health interventions are found to cost less, per DALY averted, than the relevant annual per capita gross domestic product. Box 2 illustrates this problem for diarrhoeal disease control. Making the threshold harder to meet – e.g. by only categorizing an intervention as highly cost–effective if, per DALY averted, it costs less than half of the annual per capita GDP – does not address the fundamental problem, which is that any threshold is arbitrary. More stringent thresholds would rule interventions out with as little justification as more lenient thresholds would rule them in.

Box 2 **Demonstrably effective interventions are almost certain to be cost–effective according to WHO-CHOICE: the example of diarrhoeal disease control.**

In sub-Saharan Africa, most diarrhoea-related deaths occur in children, the annual risk of death from diarrhoea in a household is often 1% or more,^9^ and 28 discounted life-years are lost per death.^10^ Thus, ignoring morbidity, the anticipated annual burden of diarrhoea can be estimated at 0.3 (0.01 × 28) disability-adjusted life-years (DALYs) per household with one child. In Kenya, a clean water intervention to reduce such deaths – e.g. chlorine or filters – could annually cost about 37 international dollars (I$) per household.^11^^,^^12^

Well-funded trials are powered to detect risk reductions of 20% or more, and particularly large trials can detect a 10% reduction.^13^^–^^15^ If we found that the clean water intervention had 20% effectiveness, implementing the intervention should avert 0.06 (0.2 × 0.3) of a DALY per household with one child. The incremental cost–effectiveness ratio, compared with doing nothing, is thus I$ 37 per 0.06  DALY averted – i.e. I$ 614 per DALY averted. At 10% effectiveness, this ratio rises to I$ 1228 per DALY averted. Both values given here for the ratio fall well below I$ 5211, which is the WHO-CHOICE threshold for a cost–effective intervention in Kenya – i.e. three times the annual per capita gross domestic product.^16^ Even if its costs were twice as high or its effectiveness were only 5% – which is probably beyond trial precision – the intervention would still be deemed cost–effective according to WHO’s criterion. Thus, if any benefit can be detected in a large trial, the intervention will be considered cost–effective.

The third limitation is the untested assumptions on which this approach is based. Social willingness to pay for health benefits is, conceptually, an appropriate way to define social value^17^ that could be informed by the results of non-market valuations based on revealed- and stated-preference approaches.^18^^,^^19^ In using a cost–effectiveness threshold that is based on a country’s per capita GDP, analysts tacitly assume that the country is willing to pay up to that threshold for the health benefit – usually without any concrete evidence of that willingness to pay. While willingness to pay for health care is related to income, there is little evidence that the relationship is linear. Other factors are also important. If averted DALYs are more highly valued in high-income countries than in low-income ones,^20^ use of cost–effectiveness thresholds based on per capita GDP per DALY averted will give a biased measure of the willingness to pay. Such thresholds will tend to be too stringent in high-income countries – thus ruling some efficient options out – and too lax in low-income countries – thus ruling some inefficient options in.

The fourth limitation is that affordability is not adequately appraised. Cost–effectiveness analyses are typically addressed to governments or international donors and aim to assist decision-making about how to spend finite budgets. Recent experience with international funding for HIV programmes may have fostered the notion that budget constraints are illusory. However, even HIV funding is less secure now than it was a few years ago.^21^^–^^25^ There is no evidence that, in the short term at least, the world will contribute the sums needed to implement all interventions that meet WHO’s criteria for cost–effectiveness. Thus, in any timeframe relevant to policy-makers, trade-offs have to be considered.

Ignoring the overall budget assigned to a health programme may be just as problematic in a high-income country as in a lower-income one – particularly for conditions that are highly prevalent. Consider a drug that adds a year to everyone’s life and costs the annual per capita GDP per person treated. Although such a drug would be categorized as highly cost–effective by WHO’s thresholds, we would have to spend the entire GDP of the country each year to give the drug to every eligible individual – i.e. to the country’s entire population.

## Benchmark interventions

Originally proposed by Weinstein and Zeckhauser,^26^ a second solution to the cost–effectiveness standard problem is to cite the cost–effectiveness of a benchmark intervention that has already been adopted in the relevant country and to use that as a threshold for acceptable cost–effectiveness. In this approach we are again using a threshold but – unlike the thresholds based on per capita GDP – this threshold is established by a retrospective analysis of existing practice.^27^ In the USA, for example, a threshold still used in cost–effectiveness analyses – US$ 50 000 per QALY gained – was based on an estimate of the cost–effectiveness of dialysis for chronic renal disease.^19^ This threshold has recently been updated to US$ 100 000 or US$ 150 000 per QALY gained.^28^ Since there is already evidence of a willingness to pay US$ 150 000 per QALY gained, it should be possible to increase overall health benefits by transferring funds from activities that cost more than this sum to activities that cost less. Thus, this approach appears to justify the adoption of any option that has a lower ICER than the benchmark.

Although such an approach may have better local relevance than thresholds based on per capita GDP, it also has substantial shortcomings. The ICER of the benchmark intervention may be a high or low outlier. For example, it may have resulted from a political decision that does not reflect the current, true measure of societal willingness to pay for health benefits. In addition, benchmarks do not take affordability into account and are not regularly updated to reflect changes in opportunity costs resulting from new technologies or delivery models, or changes in the burden of disease.

Most importantly, using a single benchmark does not address the critical question of whether there might be available options that have a better cost–effectiveness ratio than either the benchmark intervention or the intervention under evaluation. In the USA, for example, an analysis might reveal that an intervention can add a QALY for US$ 80 000 – i.e. well under the US$ 150 000 benchmark cited above. Although this would indicate that the intervention is much more cost–effective than the current benchmark, it would not tell us anything about the set of possible interventions that might add a QALY for less than US$ 80 000. Other techniques for establishing thresholds, such as human capital, contingent valuation and revealed preference approaches^26^ share the same basic strengths and weaknesses as the benchmark approach. An option to justify the one under study can almost always be found.^19^^,^^29^ One way to mitigate this problem is to consider a range of interventions adopted by public health programmes in the setting of interest and the range of ICERs from these adopted interventions. This could be achieved via a research agenda that aims to aggregate more data on willingness to pay for a unit of health benefit in a wide range of countries. In high-income countries, progress has been made on such an agenda by the translation of the available data on lives saved to data on QALYs gained.^19^

## League tables

A third approach side-steps the threshold question and focuses instead on getting the largest health impact for the budget. Conceptually, a complete set of relevant interventions would be chosen to maximize health effects. For example, if all of the interventions considered are at least somewhat scalable, they can be ranked into a so-called league table according to their ICERs.^30^ The league-table approach is based on the principle that, for any budget, health outcomes are maximized if selection of the options for implementation begins at the top of the league table – i.e. with the option with the lowest ICER – and then moves down the list, to interventions with successively higher ratios, until the budget is exhausted.^31^

Several generic league tables have been developed. WHO-CHOICE has reported simple information on the ICERs for many interventions.^3^ Separate regional league tables are available for several diseases or risk factors. For example, for the Africa D region there are tables for 60 different interventions (Table 1). Other league tables have been created for specific diseases or conditions. A 2005 article assessed the ICERs of several major HIV interventions and arranged these in a league table for sub-Saharan Africa and South-East Asia (Table 2).^33^ Other league tables are large repositories of cost–effectiveness information that can be used to assess the ranking of many interventions for wide ranges of diseases and conditions. One of the largest of these is the cost–effectiveness analysis registry maintained by Tufts Medical Center, which provides over 3600 ICERs for over 2000 health interventions.^34^

**Table 1:** A cost–effectiveness league table for malaria interventions: Africa D region^a^

Intervention (description)	Annual cost (million I$) per million people	Annual no. of DALYs averted per million people	Incremental no. of DALYs averted per million people	Incremental cost	
				Million I$ per million people	I$ per DALY averted
MAL-27 (case management with ACT, 80% coverage)^b^	0.25	26 426	26 426	0.25	9
MAL-7 (MAL-27 but 95% coverage)	0.33	31 470	5 044	0.08	16
MAL-17 (combination of ACT, IPTP and ITNs, 95% coverage)	1.07	44 115	12 645	0.74	59
MAL-20 (MAL-17 plus IRS)	1.59	49 518	5 403	0.52	96

ACT: artemisinin-based combination therapy; DALY: disability-adjusted life-year; I$: international dollars; IPTP: intermittent preventive therapy for pregnant women; IRS: indoor residual spraying; ITNs: insecticide-treated nets.

^a ^A list of countries in the Africa D region is available from: http://www.who.int/choice/demography/african_region.

^b^ The costs and DALYs averted by MAL-27 were compared with no intervention. Each of the other three options was compared with the next cheapest intervention, i.e. the intervention in the row above.

Data source: World Health Organization.^6^

**Table 2:** Example of a cost–effectiveness league table for interventions against human immunodeficiency virus infection: Africa E region^a^

Intervention (description)^b^	Annual cost, million I$	DALYs averted, millions per year	ICER, I$ per DALY averted
D1 (mass media campaign)	16	4.5	3
D2 (D1 plus peer education and treatment of sex workers for STI at 50% coverage)	57	15.6	4
D3 (D2 but 80% coverage)	79	21.3	4
D4 (D2 but 95% coverage)	89	23.8	4
D5 (D4 plus prevention, during antenatal care, of mother-to-child transmission)	249	27.3	46
D6 (D5 plus current, routine treatment of STI)	290	27.9	68
D7 (D5 plus treatment, during antenatal care, of STI)	357	28.7	80
D8 (D7 plus voluntary counselling and testing at 95% coverage)	742	30.5	220
D9 (D8 plus treatment of STI at 95% coverage)	859	30.9	290
D10 (D9 plus antiretroviral therapy with first-line drugs, without intensive monitoring)	2 125	33.2	547
D11 (D10 plus school-based education at 95% coverage)	2 202	33.3	631
D12 (D11 but with intensive monitoring)	2 350	33.4	1 144
D13 (D12 but with both first- and second-line drugs)	7 483	34.4	5 175

DALY: disability-adjusted life-year; I$: international dollars; ICER: incremental cost–effectiveness ratio; STI: sexually transmitted infections.

^a^ A list of countries in the Africa E region is available from: http://www.who.int/choice/demography/african_region.

^b^ Some packages of interventions that were more costly but less effective than those shown and those found to have higher incremental cost–effectiveness ratios than those shown were excluded from this table.

Data source: Hogan et al.^32^

A limitation of league tables is that ICERs may not be available for many relevant options or settings. Many low resource countries lack data on the costs and effectiveness of specific interventions. In these countries, the only recourse for local policy-makers is to use findings from similar countries. A bare league table omits much of the information that decision-makers might want to consider when choosing among options – e.g. the size of the affected population, whether the intervention is scalable, the health benefit per recipient and the degree of uncertainty around the ICERs.^35^^,^^36^ Perhaps, given these, we need an extended league table approach in which a list of ICERs is complemented by information on context-sensitive costs and benefits of competing options.

Against these disadvantages must be weighed several virtues. A league table indicates graduated distinctions between ICERs. Since the length of the list of interventions deemed cost–effective varies according to the budget, league tables combine considerations of cost–effectiveness with affordability.^27^ The last (least cost–effective) intervention in the table to be adopted is more likely to approximate society’s willingness to pay for health benefits than the open-ended set of commitments implied by global thresholds. Finally, league tables need not be comprehensive to support improved resource allocation. They can still indicate the potential health benefits of cancelling an existing programme and using the resources freed to fund another programme.^27^^,^^37^

## Discussion

If one intervention is deemed more cost–effective than another in the context of a fixed budget, we can say that it will yield more health benefit per unit of expenditure than that other option. However, the results of a cost–effectiveness analysis cannot indicate if an intervention is a good use of the health budget because the comparator may itself be inefficient relative to other feasible options. In addition, the notion of a fixed budget depends on the level or authority of the decision-maker. In the context of HIV treatment, for example, ICERs might indicate that viral load testing is less cost–effective than adding patients to the caseload. Although the decision-makers responsible for an HIV programme’s budgets might therefore recommend the latter approach, they might ignore – or be unaware of – the possibility that the same money spent on vaccines for childhood diseases might give greater health benefits. Funders can get a better idea of the policy relevance of the results of new cost–effectiveness analyses if they are given the ICERs for interventions that they already support. However, there is no substitute for careful reflection by policy-makers on the most efficient ways to maximize national welfare. WHO’s current cost–effectiveness thresholds can short-circuit this task, by using annual per capita GDP as a proxy for social willingness to pay.

Part of the appeal of thresholds may be the perception that cost–effectiveness analysis does not allow for fine distinctions. Rather than pretending that unrealistic precision has been achieved, thresholds have the apparent virtue of simply distinguishing interventions that meet, from those that fail to meet, a fixed criterion. It is widely acknowledged that certain aspects of cost–effectiveness theory are contentious.^31^^,^^38^^,^^39^ Practice is also imperfect and inconsistent, often making it difficult to compare results from different studies. For example, between-study variation in the selection of analytic perspective, time horizons and criteria for including or excluding particular cost components can hamper comparisons of different investigations, even when sensitivity analyses document the impact of these choices. Transparency in the assumptions made and methods used is therefore essential, as suggested by the Consolidated Health Economic Evaluation Reporting Standards.^40^ When cost–effectiveness analyses of an important policy question produce substantially different results, funders should sponsor efforts to document the source of the difference and to make appropriate adjustments, where possible.

Whether because of these uncertainties or merely for expediency, many individuals appear to believe that a statement about the ICER for an intervention – relative to a threshold based on the annual per capita GDP – is sufficient to determine cost–effectiveness. For researchers, a simple threshold removes the need to compare results to other locally relevant findings and to place their studies in context. For the editors and reviewers of journals, use of a globally accepted threshold provides reassurance that methods and results meet international norms. Use of such a threshold allows authors and reviewers to choose convenience over a more nuanced and context-specific interpretation of results. The widespread acceptance of global thresholds may thus undermine both the supply and demand for more policy-relevant analyses. On the demand side, decision-makers are offered the results of cost–effectiveness analyses that neither distinguish between programme options with widely divergent ICERs nor account for budget constraints. Decision-makers may therefore tend to dismiss cost–effectiveness analyses and revert to political or organizational interests as decision criteria. On the supply side, the availability of global cost–effectiveness thresholds undercuts the incentive of investigators to generate the nuanced, context-specific information that decision-makers need.

## Conclusion

For cost–effectiveness analyses to contribute to sound resource allocation, we argue that the estimates of both costs and effectiveness must be situated firmly within the relevant context, which includes the disease burden and budget of the setting in question. Simple cost–effectiveness thresholds – whether based on per-capita incomes or benchmark interventions – fail to evaluate and rank interventions within countries and disregard budgetary constraints. By short-circuiting a more thorough assessment of policy-relevant alternatives, they contribute little to good decision-making and can actually mislead. While the currently available data will not support comprehensive off-the-shelf league tables for most settings, the results of cost–effectiveness analyses should be compared with as many relevant interventions as reasonable in a given situation. Decision-makers would then be in a far better position to interpret the results of cost–effectiveness analyses.

A consensus process should be convened, perhaps by WHO, to develop a new framework for articulating cost–effectiveness in global health policy – specifically focusing on low- and middle-income countries. Rather than referencing a uniform standard, this new consensus should place ICERs in the context of other public health options available or already adopted in the relevant country setting – and in the context of the relevant budgets. While not resolving all of the issues affecting cost–effectiveness analysis as a guide for resource allocation, a new framework could offer an improvement on the use of simple thresholds based on per-capita incomes.
